# Oleuropein-Rich Gellan Gum/Alginate Films as Innovative Treatments against Photo-Induced Skin Aging

**DOI:** 10.3390/molecules28114352

**Published:** 2023-05-25

**Authors:** Francesco Busto, Caterina Licini, Alessia Luccarini, Elisabetta Damiani, Monica Mattioli-Belmonte, Stefania Cometa, Elvira De Giglio

**Affiliations:** 1 Department of Chemistry, University of Bari, Via Orabona 4, 70126 Bari, Italy; f.busto3@studenti.uniba.it; 2 INSTM, National Consortium of Materials Science and Technology, Via G. Giusti 9, 50121 Florence, Italy; m.mattioli@staff.univpm.it; 3 Department of Clinical and Molecular Sciences, Università Politecnica delle Marche, Via Tronto 10/a, 60126 Ancona, Italy; c.licini@staff.univpm.it; 4 Department of Life and Environmental Sciences, Università Politecnica delle Marche, Via Brecce Bianche, 60131 Ancona, Italy; a.luccarini@pm.univpm.it (A.L.); e.damiani@univpm.it (E.D.); 5 Jaber Innovation s.r.l., Via Calcutta 8, 00144 Rome, Italy

**Keywords:** oleuropein, gellan gum, alginate, facial masks, UVA-induced damage, aged skin, Franz cell, normal human dermal fibroblasts

## Abstract

*Olea europaea* L. leaf extracts (OLEs) represent highly value-added agro-industrial byproducts, being promising sources of significant antioxidant compounds, such as their main component, oleuropein. In this work, hydrogel films based on low-acyl gellan gum (GG) blended with sodium alginate (NaALG) were loaded with OLE and crosslinked with tartaric acid (TA). The films’ ability to act as an antioxidant and photoprotectant against UVA-induced photoaging, thanks to their capability to convey oleuropein to the skin, were examined with the aim of a potential application as facial masks. Biological in vitro performances of the proposed materials were tested on normal human dermal fibroblasts (NhDFs), both under normal conditions and after aging-induced UVA treatment. Overall, our results clearly show the intriguing properties of the proposed hydrogels as effective and fully naturally formulated anti-photoaging smart materials for potential use as facial masks.

## 1. Introduction

Facial masks represent one of the most promising cosmetic devices utilized for skin hydration, rejuvenation, lightening, etc. They are ideal for topical active ingredient delivery since the polymeric matrix of facial masks can be loaded directly onto a defined area of the face and can have a longer residence time than classical cosmetic formulations, such as creams and gels. The main purpose of facial masks is to enhance skin hydration and provide the skin with beneficial effects exerted by the active ingredients contained in the mask. As reported by Nilforoushzadeh et al. [1], facial masks can be classified into (i) sheet masks, (ii) peel-off masks, (iii) rinse-off masks and (iv) hydrogels. Hydrogels are often the preferred choice, due to their high hydrophilic nature and affinity with the skin, high water content and high ability in delivering active ingredients to the skin [2]. Hydrogels, in addition to possessing superior moisturizing properties, show good elasticity and adaptability to facial skin, guaranteeing superior compliance with respect to other facial mask types. Usually, hydrogel facial masks are based on polyvinyl alcohol (PVA) or polyvinyl acetate (PVAc), due to their optimal physical and mechanical properties. However, PVA-based masks could result particularly occlusive [3].

Natural polymers and, in particular, polysaccharide-based hydrogels are considered highly promising alternative materials for facial masks showing the required chemical, physical and mechanical properties in addition to low toxicity, high biocompatibility and biodegradability. Among the different polysaccharides, gellan gum (GG), an anionic, water-soluble, extracellular non-branched polysaccharide produced via bacterial fermentation, demonstrated to possess many advantageous properties, such as ductility, thermo-responsibility, non-toxicity and biocompatibility. GG has been proposed in wound dressing [4], transdermal drug delivery [5], skin burn wound healing [6], skin tissue engineering [7], etc. Moreover, it has been widely used in cosmetics, such as shampoos, body washes, sprayable sunscreens, etc., as well as an emulsion stabilizer, through preventing the separation of oil and water, or as a suspension agent for poorly soluble active ingredients [8].

Sodium alginate (NaALG) is a brown algae-extracted polysaccharide, similar in utility profile to GG and well-known to be biocompatible, hydrophilic, relatively economical and mostly used in the pharmaceutical and food industry. Differently from GG, NaALG has been frequently proposed as an ingredient for making facial masks in different patents [9,10] and commercial products (for example, those produced by Biogenie Beaute Concept (Johor Bahru, Johor, Malaysia) or Magiray Israel Cosmetics (Tel Aviv, Israel)). On the other hand, even if GG alone can be easily processed into transparent hydrogel sheets, when this material is crosslinked, corrugated and crumpled films are obtained.

Therefore, in this work, we decided to test optimized blends of GG/NaALG to obtain stable, biocompatible, easy-to-apply and sufficiently adhesive films able to provide a pleasant skin feeling and a smooth and uniform texture for potential use as facial masks. Recently, Reczynska-Kolman and coworkers proposed GG and a mixture of GG and NaALG, containing lipid nanoparticles loaded with the antibacterial peptide nisin, for wound healing application and the treatment of bacterial infections in wounds [11]. However, no examples of GG or GG/NaALG hydrogel films for facial mask applications are present in the literature.

It is well known that both GG and NaALG can form hydrogels in the presence of cations (especially divalent ones), due to the electrostatic interactions of these ions with the carboxylate groups of the polymer chains. In this study, a covalent crosslinking was chosen to supply superior stability in a highly moisture-rich ambient environment. A biobased carboxylic diacid such as tartaric acid (TA) was chosen as an easily accessible, cost-effective and green crosslinking agent [12].

The obtained films were formulated to counteract the harmful effects of ultraviolet (UV) radiation on the skin, in particular, via UVA (320–400 nm) exposure of the dermis. UVA accounts for 95% of solar UV light and is more penetrating than UVB, reaching the dermal layer. This extrinsic factor, together with intrinsic ones, stimulates cumulative skin damage leading to photoaging. Deep wrinkles and skin laxity are the macroscopical consequences of photoaging, mainly associated with changes in fibrillar and amorphous constituents of the dermal extracellular matrix (ECM) and alterations in fibroblasts [13,14,15]. Furthermore, UVA generates an inflammatory response via the production of several pro-inflammatory mediators, such as Tumor Necrosis Factor-α (TNF-α) and Interleukin-6 (IL-6) [16,17]. In this context, the use of plant-derived, polyphenol-rich, safe and effective antioxidant ingredients to reduce the consequences of UVA exposure could be an appropriate strategy to ameliorate the state of mature skin, counteracting the damaging effects produced through UV radiation exposure. Agro-industrial byproducts and their major bioactive compounds could be potentially used for skin photoprotection. In this respect, olive leaf extract (OLE) represents an agro-industrial byproduct that is rich in oleuropein, a phenol that has been already demonstrated to act as a free radical scavenger and to protect the skin against UVB-induced damage, suppressing intracellular ROS levels and oxidation of proteins through inducing conformational changes in proteasome activity [18]. Moreover, an OLE supplement was shown to reduce UV-induced erythema and tanning [19], and its topical application indicated photoprotective and antioxidant features in association with organic UV filters in sunscreen formulations [20]. This finding was also observed by Jesus and coworkers, who reviewed a series of synthetic and natural compounds having promising photoprotection properties for the formulation of next-generation UV filters [21]. Among these compounds, the use of active ingredients derived from agri-food wastes, such as oleuropein-rich OLE, perfectly responds to the need for applying sustainable practices and circular economy concepts [22].

In this work, we hypothesized that facial mask devices containing OLE could protect skin against UVA-induced damage after directly applying the mask on the skin and releasing oleuropein in situ. The novelty of our study consists in proposing GG/NaALG crosslinked blends as films particularly compliant as facial masks, as well as the loading of an olive leaf extract particularly rich in oleuropein.

Chemical–physical characterizations of the proposed materials were carried out by means of Fourier-transform infrared spectroscopy in attenuated transmittance reflection mode (FTIR/ATR), X-ray photoelectron spectroscopy (XPS), swelling tests, gel fraction, water holding capacity tests and thermogravimetric analysis (TGA). The total polyphenol content (TPC) and antioxidant activity of the extract, as well as the extract-loaded films, were investigated through Folin–Ciocâlteu’s reagent and DPPH assays, respectively. Finally, the ability of the film to convey oleuropein to the skin was evaluated using a Franz cell.

An in vitro study with normal human dermal fibroblasts (NhDFs), both under normal conditions and after UVA-induced damage, was carried out to investigate the viability and morphology of the cells and the expression of ECM components, such as type I collagen and decorin. MTT assay, F-actin staining, live cell imaging and Western blotting were conducted for these purposes. Moreover, we examined the possible anti-inflammatory effect of the developed systems on UVA-irradiated NhDFs, investigating pNF-kB expression and TNF-α and IL-6 mRNA levels.

## 2. Results and Discussion

### 2.1. Chemical–Physical Characterization

In this work, GG/NaALG films were developed for a potential application as facial masks able to release oleuropein-rich olive leaf extract to the skin. As reported in Section 3.2 and in the Supplementary Materials (see Figure S1), a preliminary investigation on the best GG/NaALG mass ratio was carried out, considering the oleuropein release from the films. These preliminary tests showed that the best release performances occurred using films having higher GG content, even if the presence of NaALG was essential to obtain smoother and more homogeneous structures. Therefore, two composite films were chosen, i.e., GG1.6NaALG0.4 and GG1.2NaALG0.8, and analyzed in terms of chemical–physical properties and biological performance.

**Figure 1:**
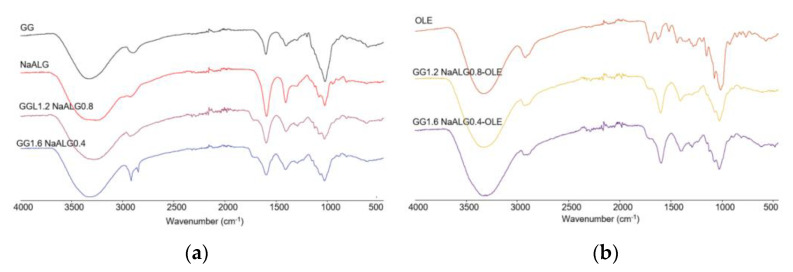
FT-IR/ATR analyses of (**a**) GG, NaALG, GG1.2NaALG0.8 and GG1.6NaALG0.4 and (**b**) OLE, GG1.2NaALG0.8-OLE and GG1.6NaALG0.4-OLE films.

First of all, FT-IR analysis in ATR mode on the investigated films (both unloaded and OLE-loaded ones) as well as on their feedstocks was carried out, as shown in Figure 1, to study possible interactions between the two polymers and/or between polymers and the natural extract.

In panel a, a comparison of pure GG and NaALG with the two films (i.e., GG1.2NaALG0.8 and GG1.6NaALG0.4) was shown. As observed, all the main characteristic bands of GG and NaALG are present in the films. The bands at 3331 and 3267 cm^−1^ can be ascribed to OH stretching vibrations in pure GG and the NaALG backbone, respectively. These bands fall at 3275 and 3280 cm^−1^ in GG1.2NaALG0.8 and GG1.6NaALG0.4, respectively. The wavenumber decrease of the OH stretching band could be ascribed to a higher intermolecular interaction which can occur between NaALG and GG chains, mainly based on hydrogen bonds, as this is well-documented in the literature [14,15]. The bands at 2850 and 2918 cm^−1^ are related to the CH stretching vibration of methyl and methylene in both carbohydrates. The bands at 1594 and 1407 cm^−1^ of the pure NaALG correspond to the asymmetric and symmetric stretching peaks of the COONa group, respectively. Carboxyl groups are also present in the structure of GG, with the characteristic bands falling at almost similar wavenumbers (i.e., 1601 and 1404 cm^−1^) [14,16]. In GG1.2NaALG0.8 and GG1.6NaALG0.4, these bands fall at 1598 and 1408 cm^−1^ and at 1597 and 1403 cm^−1^, respectively. The bands at around 1716 cm^−1^ are due to carbonyl (C=O) stretching of TA and are also present in the crosslinked hydrogel films, due to the ester groups formed, as already reported by Singh et al. [12] and Fahmi et al. [23].

In panel b, OLE, GG1.2NaALG0.8-OLE and GG1.6NaALG0.4-OLE spectra are reported. The main bands observed in OLE fall at 3320 and 1703 cm^−1^ and could be related to the stretching of the O–H and C=O groups, present in different chemical compounds of olive leaf (such as oleuropein, apigenin-7-glucoside and/or luteolin-7-glucoside), as reported by Khalil et al. [24]. These absorptions overlap with those already present in the GG/NaALG-based films, even if in both OLE-containing films the C=O stretching peak falls at slightly lower wavenumbers (i.e., 1710 cm^−1^).

**Figure 2:**
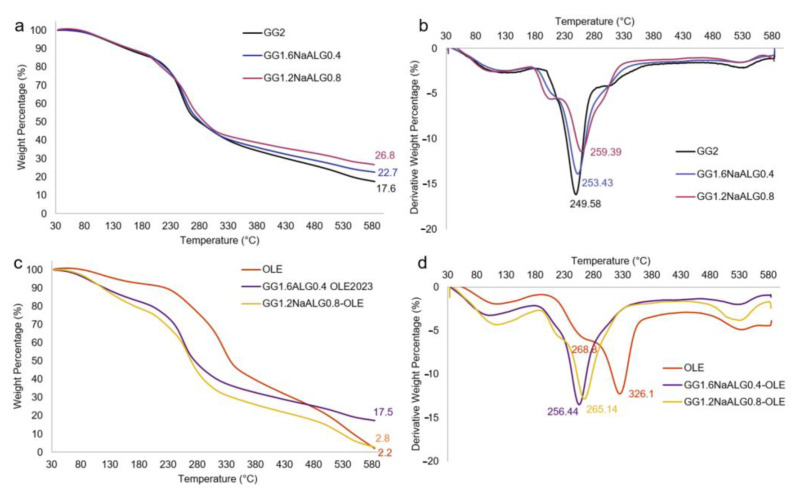
Thermograms (**a**,**c**) and derivative traces (**b**,**d**) of (**a**) GG2, GG1.6NaALG0.4 and GG1.2NaALG0.8 films and (**b**) OLE, GG1.6NaALG0.4-OLE and GG1.2NaALG0.8-OLE films.

TGA analyses were carried out in order to shed light on the stability of the films as well as on the possible interactions between GG and NaALG or between polymers and natural extract. Firstly, TGA analyses were carried out on GG and NaALG powders (traces reported in Figure S2 of Supplementary Data). Pure GG powder presented a main decomposition step with a sharp derivative peak centered at 265 °C, with a final residue of 16.4%. Pure NaALG presented a main decomposition step with a large derivative peak centered at 248.7 °C, thus showing lower thermal stability and a higher residue (i.e., 33.4%) than GG, as already reported in the literature [25]. For the TA-crosslinked films reported in Figure 2a,b, it can be observed that the higher the gellan content in the films, the lower the residue at 600 °C, as expected. This is also demonstrated via the analysis carried out on a film crosslinked with TA based only on GG, which presented the lowest residue. Interestingly, when GG was crosslinked with TA (sample GG2), the main decomposition peak fell to 249.6 °C (hence a destabilization of the polymer of about 15 °C is observed), with a shoulder at higher temperatures. When NaALG was present in the films, the appearance of a new decomposition peak at about 210 °C was observed, more intense at higher NaALG content. The GG1.2NaALG0.8 film presented the main decomposition peak centered at higher temperatures (i.e., 259.4 °C) than in GG1.6NaALG0.4 and GG2 films (i.e., 253.4 and 249.6 °C, respectively). This indicates that the presence of NaALG in the system leads to less destabilization of the GG, probably due to the interaction between GG and NaALG chains, as already demonstrated via FT-IR analysis. This result is in agreement with what was observed by Naji-Tabasi et al., who found that the thermal stability of the hydrogels increased when combining the two polymers [15].

Finally, in panels c and d, thermograms and derivative thermograms of OLE and OLE-containing films are reported.

For OLE, three stages of degradation at 268.8, 326.1 and 534.5 °C, excluding the first stage due to the elimination of water and volatiles, can be ascribed to the degradation of hemicellulose, lignin, wax, etc. present in the natural extract, including oleuropein and other active substances. When OLE was included into the film formulations, no significant changes in the thermal decomposition stages with respect to the bare films were observed, except for the main decomposition peak, which shifted at a higher temperature for both films: +3 and +5.7 °C for the GG1.2NaALG0.8 and GG1.6NaALG0.4-based films, respectively. The absence of decomposition peaks relevant to OLE could be related to the protection of the extract from decomposition exerted by the polymer matrix, as already evidenced by Fabiano et al. [26].

In Table 1, the surface atomic compositions of GG, NaALG, OLE and films with or without the plant extract are reported. As expected, sodium, present mainly in NaALG, was more abundant in GG1.2NaALG0.8 than in GG1.6NaALG0.4 films, while nitrogen, present only in GG, was more abundant in GG1.6NaALG0.4 than in GG1.2NaALG0.8 surfaces. Moreover, it can be observed that silicon, related to the presence of OLE, was also present in the OLE-loaded films. In conclusion, the surface atomic composition substantially reflects the expected bulk composition of the materials.

Moreover, OLE, GG and NaALG raw materials were analyzed more in depth, considering the C1s signals and the relevant curve fittings (see Figure S3 in the Supplementary Material section). Aliphatic (CHx), hydroxy/ether (C-OR), emiacetalic/keto (O-CO/C=O) and carboxylic/ester (COOH/COOR) groups are present both in the polymers and in the olive extract.

In the latter, aromatic groups were also detected but their binding energies overlapped with those of the aliphatic ones, whereas a carbon in α to a carboxylic/ester groups was present, differently from the polymer matrices.

**Figure 3:**
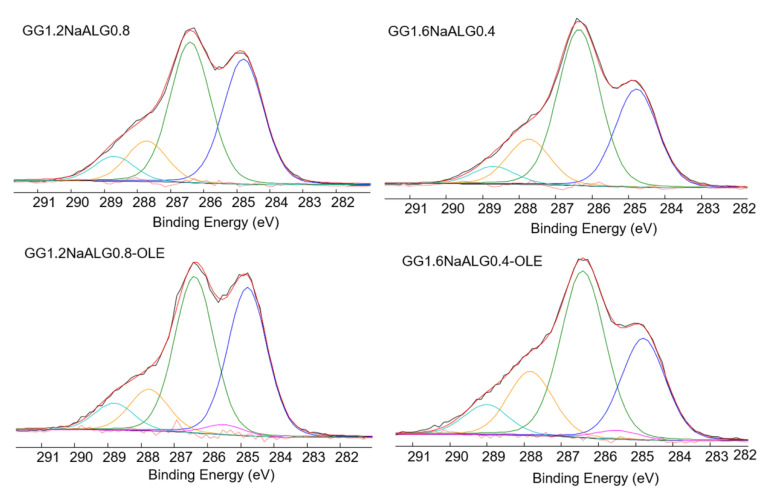
C1s high-resolution spectra and relevant curve fittings recorded on GG1.2NaALG0.8, GG1.6NaALG0.4 and the same films loaded with OLE.

Indeed, in the C1s curve fittings of the films, reported in Figure 3, the main difference between the films with and without OLE was related to the presence of this C-COOR peak. No additional speculations can be made on the other contributions since the presence of OLE contributed to all other peaks (see Table 2).

### 2.2. Hydrogel Films Swelling, Gel Fraction and Water Vapor Transmission Rate (WVTR)

A facial mask is usually employed in its swollen state; therefore, commercial products are supplied appropriately hydrated. In this case, a preservative must be added to guarantee protection against bacterial contamination. Here, we proposed hydrogels in the dry state but which are easily and rapidly swellable, thus avoiding the employment of preservatives. The hydrogel-swelling kinetics were accurately studied, as well as the gel fraction, i.e., the non-soluble polymer fraction due to the cross-linking process. The gel fraction gives an indication of the hydrogel stability.

**Figure 4:**
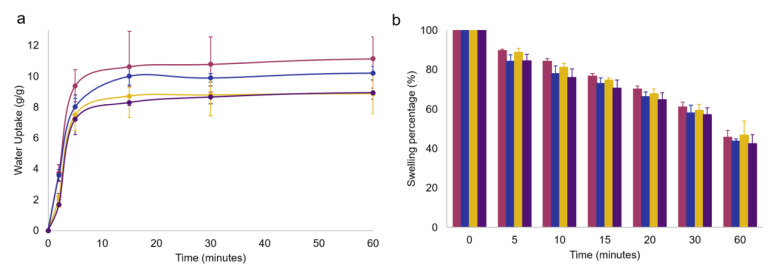
(**a**) Swelling kinetics in PBS at 32 °C and (**b**) deswelling kinetics up to 1 h of GG1.2NaALG0.8 (magenta), GG1.6NaALG0.4 (dark blue), GG1.2NaALG0.8-OLE (ochre) and GG1.6NaALG0.4-OLE (dark violet) hydrogel films. Error bars obtained as standard deviation over three replicates.

In Figure 4a, the swelling kinetics of GG1.2NaALG0.8, GG1.6NaALG0.4 and the relevant OLE-loaded hydrogels are reported. It is important to underline that very fast swelling kinetics were obtained for both OLE-containing films. The experiment was carried out in PBS at 32 °C up to 24 h but, in the figure, a time range of 60 min is reported, since a plateau was reached just after 15 min.

The recorded water uptake values for OLE-free films settled at around 10 g/g, agreed with those reported by Chen and coworkers, who developed gellan/alginate-based hydrogel scaffolds for osteochondral repair applications [27]. Visual examination of the films indicated that the samples containing a higher percentage of GG are better able to maintain integrity when swollen, while those containing a lower GG amount are much more susceptible to breakage. The presence of OLE caused a slight water uptake decrease: this could be ascribed to the simultaneous OLE release, as well as to the presence of a non-swellable component, such as OLE, in the polymer network.

In Figure 4b, a simulation of the room-temperature drying of the hydrogel films was performed, showing, for all the systems, high maintenance of hydration: after 15 min, a typical exposure time of a facial mask, a swelling percentage ≥ 70% was still recorded, indicating the absence of excessive film drying which could lead to a sensation of skin tightness.

Moreover, gel fraction and water holding capacity (WHC) tests were carried out and the results are reported in Table 3.

Gel fraction tests showed superior stability of the samples with a higher GG amount. Moreover, gel fraction tests carried out on uncrosslinked films indicate an almost total solubilization of the films in water at the end of the test, demonstrating the need of the crosslinking process to obtain stable structures.

WHC% results demonstrate a high water retention capacity for all the films, regardless of the presence of the olive leaf extract. These values reflect the effectiveness of the crosslinking reaction. On the other hand, irrespective of the presence of OLE in the films, higher percentages of GG in composites seem to lead to a higher water holding capacity. This is in agreement with the superior gel hardness of the films with higher GG amount, as also observed by Naji-Tabasi and coworkers in their freeze-dried Ca^2+^-crosslinked gellan–alginate hydrogels [15].

### 2.3. Total Polyphenol Content (TPC) and Radical Scavenging Activity (RSA%) Results

**Figure 5:**
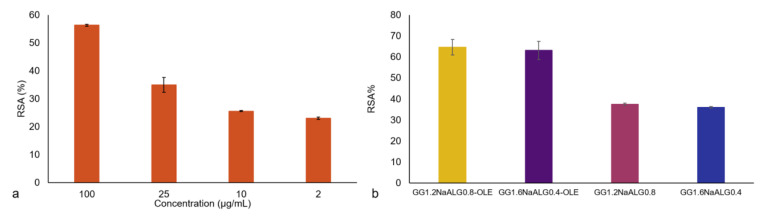
Antioxidant activity of OLE (**a**) and of films with and without OLE (**b**) evaluated via the DPPH assay.

The evaluation of TPC was carried out on the employed OLE and the obtained value, expressed as mg of gallic acid equivalent to g of dry extract, was 130.4 GAE/g. This value is in good agreement with what is reported in the literature [26,28,29], taking into account that the observed differences can be ascribable to the natural product’s variability. Indeed, the employed OLE showed good antioxidant activity as detected with the DPPH assay (Figure 5a). The percentage of radical scavenging activity increased with OLE concentration, as expected, showing an RSA (%) equal to 56.3 ± 0.3% at 100 μg/mL.

Concerning the antioxidant performance of the films, an interesting activity was also recorded in the unloaded materials, probably due to a radical scavenging ability exerted by some components of the films. In particular, GG showed negligible antioxidant properties [30] while NaALG was found to have mild antioxidant properties [31]. On the other hand, significant antioxidant activity can be associated with tartaric acid used as crosslinker [32,33]. Indeed, a solution of TA 100 µg/mL was tested and an RSA% value equal to 41.0 ± 1.2% was found. The films’ antioxidant activity was significantly enhanced by the presence of OLE in both films, reaching an RSA% of 65 ± 4% and 63 ± 4% for GG1.2NaALG0.8-OLE and GG1.6NaALG0.4-OLE films, respectively (see Figure 5b).

Additionally, TPC was also evaluated in the films. TPC equal to 76 and 49 GAE/g for GG1.2NaALG0.8-OLE and GG1.6NaALG0.4-OLE films were obtained, respectively. On the contrary, negligible TPC was detected in both OLE-free films.

### 2.4. Oleuropein Detection via HPLC and Franz Cell Results

HPLC analysis was employed to detect oleuropein both in the extract and in the prepared film formulations. Oleuropein was chosen since it is the most abundant molecule present in this plant extract (24.7 ± 0.3%).

Firstly, a direct oleuropein release was monitored through immersing pieces of films in PBS for up to 24 h. This procedure was initially used to choose the best GG/NaALG ratios (see Section 3.2 and Figure S3) for the GG/NaALG-based films, where the highest oleuropein release was obtained from GG1.2NaALG0.8 and GG1.6NaALG0.4 ones (i.e., 81 ± 7 and 74 ± 9%). The systems containing GG0.4NaALG1.6 and GG0.8NaALG1.2 released lower amounts of oleuropein (i.e., 43 ± 3 and 47 ± 4%, respectively); therefore, these systems were no longer considered for further investigations.

As far as the in vitro skin permeation studies are concerned, no transdermal oleuropein release was observed for up to 2 h, but 28 ± 8 and 31 ± 6% of the total oleuropein present in GG1.2NaALG0.8 and GG1.6NaALG0.4, respectively, was detected on the membrane simulating skin after immersion of the membrane in PBS. Moreover, a TPC test, performed on the same StratM^®^ membranes in contact with GG1.2NaALG0.8 and GG1.6NaALG0.4 films, showed a TPC equal to 37 and 39 GAE/g, respectively. Franz cell experiments showed that after 2 h, a permeation of phenols equal to 45 GAE/g for both film formulations was detected, even if this time is not compatible with the potential use of these films as facial masks.

Overall, the results obtained clearly suggest that these oleuropein-rich materials have potential as possible topical skin treatments capable of reducing skin oxidative damage such as that related to UV exposure.

### 2.5. Cell Viability

**Figure 6:**
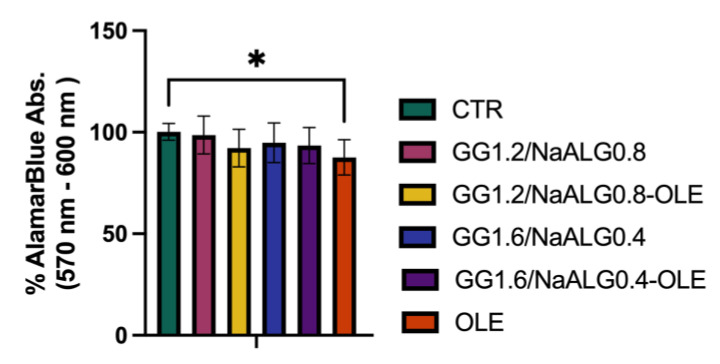
Histograms representing viability percentage in differently treated NhDFs at 24 h. (One-way ANOVA: *p* value = 0.05; * *p* ≤ 0.05).

To test the cytotoxicity of the different hydrogels (GG1.2NaALG0.8, GG1.6NaALG0.4, GG1.2NaALG0.8-OLE, GG1.6NaALG0.4-OLE), these materials were incubated in normal medium for 24 h at 37 °C to obtain conditioned media (CM), as described in the Section 3. We observed that after 24 h of cell treatment with the CM, all the systems exerted no significant effects on the NhDF’s viability, suggesting that GG1.2NaALG0.8 and GG1.6NaALG0.4, with or without OLE, are not cytotoxic to NhDFs. Interestingly, we observed a slight albeit significant decrease in cell viability after 24 h incubation with OLE extract, indicating that the developed systems are able to convey OLE, reducing its negative effect (Figure 6).

**Figure 7:**
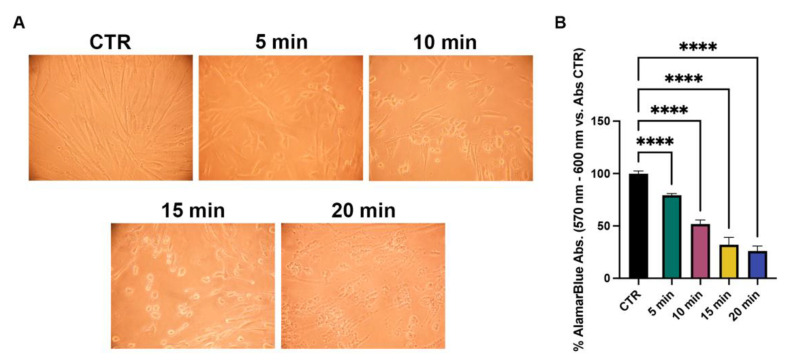
Evaluation of different UVA dose effects on NhDF cells. (**A**) Phase-contrast images and (**B**) viability percentages of NhDFs after 5, 10, 15 and 20 min of UVA irradiation. (One-way ANOVA: *p* value ≤ 0.0001; **** *p* ≤ 0.0001).

After the non-toxic effect was assessed, NhDFs were exposed to increasing UVA irradiation times (5, 10, 15 and 20 min) to test the appropriate exposure for the subsequent experiments. Based on the viability results (30% reduction in cell viability) and morphological changes, a UVA exposure of 5 min (~13.5 J/cm^2^) was chosen (Figure 7).

### 2.6. Morphological Features and Live Cell Imaging in NhDFs

In normal skin, fibroblasts are characterized by a typical elongated shape, whilst reduced size is one of the key features of senescent fibroblasts in photoaged dermis [14]. At this regard, cell morphology and area percentage were evaluated to investigate the influence of the developed systems on this fibroblast aspect.

**Figure 8:**
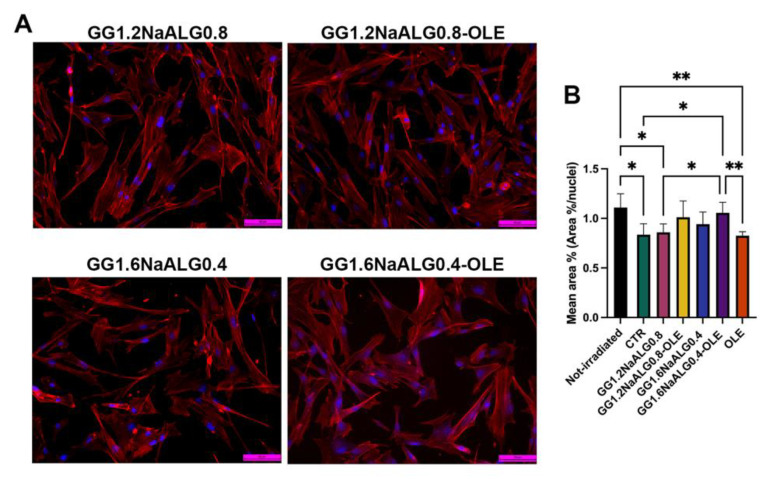
Morphological evaluation of NhDFs at 24 h after UVA irradiation. (**A**) F-actin staining of treated NhDFs (magnification: 20×; scale bar: 50 μm). (**B**) Histogram representing the mean area percentage of NhDFs. (One-way ANOVA: *p* = 0.002; * *p* ≤ 0.05; ** *p* ≤ 0.01.)

Non-irradiated NhDFs showed a normal spindle morphology, with evident filopodia and lamellipodia (Figure S4). After UVA irradiation, most of the cells cultivated in normal medium appeared shorter and wider compared to the non-irradiated ones, exhibiting a rhomboid morphology and lower area percentage (Figure S4 and Figure 8). After the treatments with the CM from GG1.2NaALG0.8 and GG1.6NaALG0.4, several cells appeared more elongated, although the area percentage did not change compared to the area of the non-treated ones (Figure 8). The addition of OLE to the materials restored spindle morphology in the majority of fibroblasts and, in the case of GG1.6NaALG0.4-OLE, re-established the area percentage at values very close to those measured in non-irradiated cells (Figure 8).

4D cell imaging was conducted via holotomographic microscopy allowing to observe cell behavior in response to UVA irradiation and incubation with the CM from GG1.2NaALG0.8-OLE and GG1.6NaALG0.4-OLE for 24 h. Non-irradiated fibroblasts and UVA-irradiated cells cultured in normal media were used as references. Non-irradiated fibroblasts showed elongated morphology and high mobility, and several mitoses were observed during the 24 h (Supplementary Video S1). On the contrary, UVA-irradiated cells were smaller and compact, with low mobility. During the 24 h, we observed cells undergoing apoptosis (Supplementary Video S2).

During treatment with the CM from GG1.2NaALG0.8-OLE, some UVA-irradiated fibroblasts regained mobility during the early hours of incubation and elongated morphology from the fifth hour of treatment. (Supplementary Video S3). On the other hand, all UVA-irradiated NhDFs treated with GG1.6NaALG0.4-OLE regained their mobility and elongated morphology after 8 h (Supplementary Video S4), and after 20 h, all the cells showed movement and morphology similar to the non-irradiated ones.

Fibroblast size reduction observed in photoaged dermis is one of the most important consequences of UVA irradiation. This event is also correlated with other several effects, such as the decreased production of ECM components, the regulation of ECM degradation, and the increase in mitochondrial ROS generation [14]. Our results suggested that OLE-loaded hydrogels, particularly GG1.6NaALG0.4-OLE, were able to restore the normal morphology in UVA-exposed fibroblasts, contrasting this fundamental aspect of photoaged skin.

### 2.7. Assessment of Anti-Oxidant Effects in NhDFs

Oxidative stress can be considered as a mechanism that leads to inflammation and tissue damage, one of the causes of photoaging inflicted by exposure of skin to UVA radiation [34,35].

Previous studies have demonstrated the protective role that OLE exerts against oxidative stress in UV-irradiated cells [21,36,37], and these are now further supported by our results that demonstrate the antioxidant activity of the developed materials using the DPPH assay (see Figure 5b).

**Figure 9:**
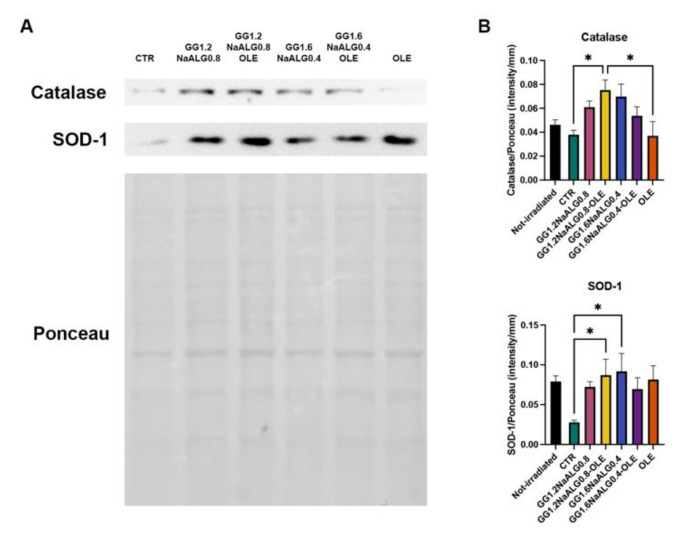
Western blotting for catalase and SOD-1 in UVA-exposed NhDFs. (**A**) Representative blot and Ponceau staining. (**B**) Histograms of densitometric analysis (one-way ANOVA for catalase and SOD-1: *p* ≤ 0.05; * *p* ≤ 0.05).

In NhDFs treated with all the CM, we observed an increasing trend of the expression of two proteins involved in cell defense against oxidative stress, catalase and superoxide dismutase 1 (SOD-1). Particularly, their expression was significantly improved in cells treated with GG1.2NaALG0.8 CM compared to cells cultivated in NM (Figure 9).

Our results indicate that the developed materials, especially GG1.2NaALG0.8, are capable of exerting a protective activity against oxidation through modulating the expression of catalase and SOD-1, which inactivate oxidation products [13]. It is well known that ROS production following UVA irradiation is also implicated in the inflammatory response and the modulation of ECM breakdown [14]. For this reason, the antioxidant effect observed after GG1.2NaALG0.8 CM treatment could be a key point of the photoprotective effects in UVA-irradiated NhDFs.

### 2.8. Assessment of Hydrogel Effects on ECM Protein Production in NhDFs

**Figure 10:**
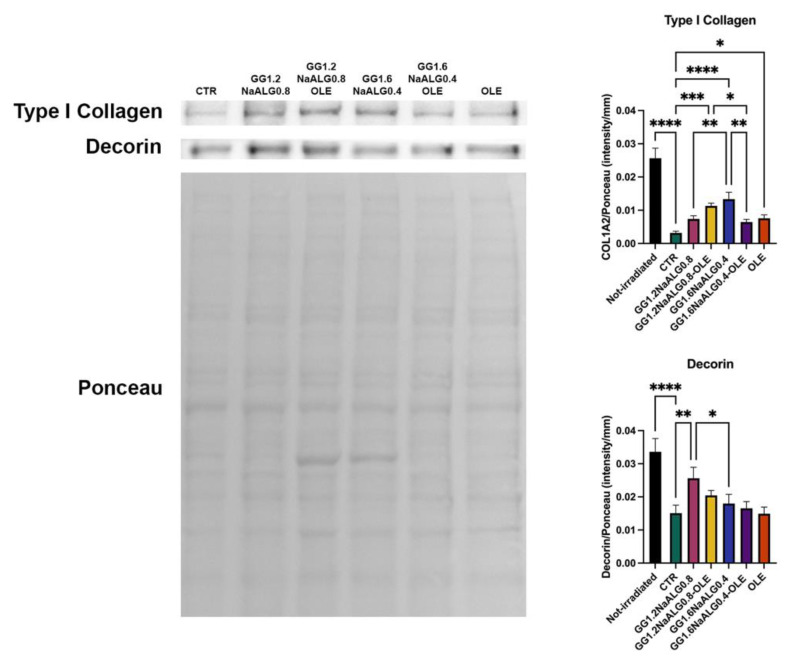
Western blotting for type I collagen and decorin in UVA-exposed NhDFs. Representative blot and Ponceau staining. Histograms of densitometric analysis (one-way ANOVA for both proteins: *p* ≤ 0.0001; * *p* ≤ 0.05; ** *p* ≤ 0.01; *** *p* ≤ 0.001; **** *p* ≤ 0.0001).

Dermal ECM is composed of fibrillar and amorphous components. Among them, type I collagen and decorin represent two main targets of photoaging. type I collagen constitutes about 80% of total collagen, while decorin is the predominant proteoglycan in human dermis, with a pivotal role in the regulation of collagen fibrillogenesis and in the maintenance of mechanical properties of collagen fibrils. The interaction between these proteins provides skin structural integrity and appropriate tensile strength [14,38,39]. Type I collagen and decorin expression decreased drastically in irradiated control cells with respect to the non-irradiated ones (Figure 10). The CM obtained from the different materials, also modulated via OLE availability, was capable of reducing the inhibition of these two proteins in fibroblasts caused by UVA exposure (Figure 10).

Overall, the results demonstrate that GG1.2NaALG0.8 and GG1.2NaALG0.8-OLE hydrogels are able to counteract the decrease in type I collagen and decorin production in UVA-irradiated fibroblasts, a typical event occurring in aged and photoaged skin [14,38].

### 2.9. Regulation of Inflammatory Markers in UVA-Irradiated NhDFs

RT PCR was performed to reveal the mRNA expression of TNF-α and IL-6.

TNF-α and IL-6 play a key role in photoaging. Following UV exposure, immune and non-immune cells in the skin start to produce TNF-α and IL-6 cytokines, as consequence of inflammation triggering. Concerning fibroblasts, increased expression of these molecules is implicated in the production of metalloproteases and other enzymes (i.e., cathepsin K), which are the main actors in the degradation of fibrillar components and proteoglycans in ECM. Moreover, TNF-α also inhibits proteoglycan biosynthesis [40].

We noted an increase in the inflammatory TNF-α and IL-6 mRNAs in UVA-irradiated (CTR) cells compared with the non-irradiated ones, albeit the observed differences were not statistically significant.

**Figure 11:**
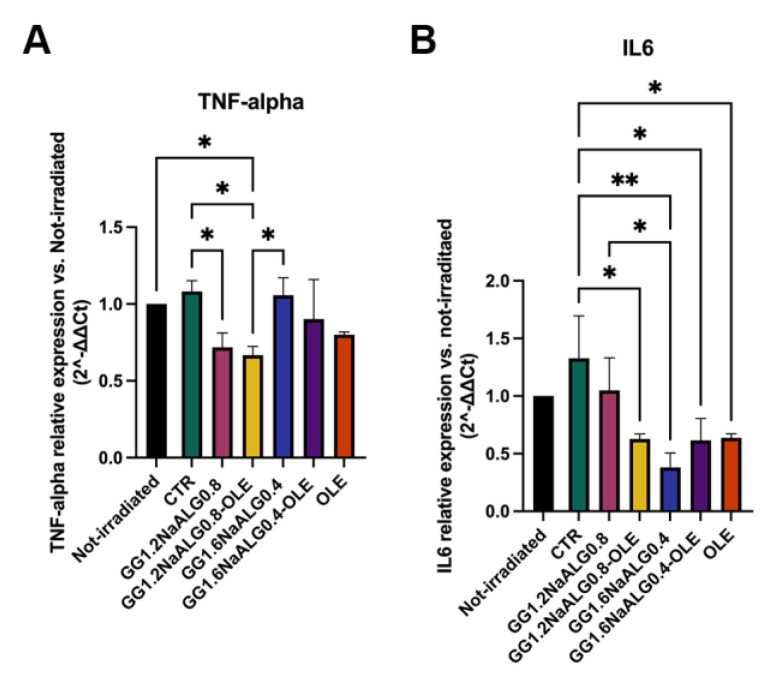
Histograms representing TNF-α (**A**) and IL-6 (**B**) mRNA levels in UVA-irradiated NhDFs. (One-way ANOVA: TNF-α *p* = 0.0004, IL-6 *p* < 0.0001; * *p* ≤ 0.05, ** *p* ≤ 0.01.)

All the treatments were effective in reducing the inflammatory molecules in UVA-irradiated fibroblasts (Figure 11). In particular, the treatment with the CM from GG1.2NaALG0.8-OLE successfully reduced the expression of both mRNAs, compared to non-treated cells (CTR). Furthermore, cells treated with GG1.2NaALG0.8 CM showed a decrease in TNF-α expression, but not in IL-6, suggesting that this hydrogel improved its anti-inflammatory ability once loaded with OLE. Both GG1.6NaALG0.4 and GG1.6NaALG0.4-OLE favored the reduction of only IL-6.

Overall, the GG1.2NaALG0.8-OLE hydrogel appears to be the best one in view of the decrease in both TNF-α and IL-6 levels, contrasting UVA-triggered inflammation, thus providing further support in favor of its anti-photoaging effects for skin applications.

## 3. Materials and Methods

### 3.1. Materials

Low-acyl gellan gum (Phytagel™, molecular weight 1 × 10^6^ g/mol), coded as GG, sodium alginate (Cat #180947, Sigma-Aldrich, St. Louis, MO, USA, viscosity 15–25 cP, 1% in H_2_O), coded as NaALG, tartaric acid (TA) and sodium hypophosphite (SHP) have been purchased from Sigma-Aldrich (Merck, Milan, Italy). All reagents and materials have been used as received without further purification. *Olea europaea* L. leaf extracts (OLEs), containing oleuropein > 20%, were kindly supplied by EVRA s.r.l. (Italy).

### 3.2. GG/NaALG Hydrogel Films Preparation

To prepare GG/NaALG hydrogel films, different ratios of GG/NaALG were tested, maintaining the sum of the two polymer percentages equal to 2% (*w*/*v*) in distilled water at 60 °C under continuous stirring on a magnetic plate (VELP Scientifica Srl, Usmate, Italy). TA was initially solubilized in distilled water, followed by the polymer powder addition. The crosslinking procedure was optimized starting from that reported for NaALG by Singh et al. [12], making several modifications in terms of temperature, times, employment of the catalyst, TA concentrations as well as the GG/NaALG mass ratio, as detailed in the Supplementary Materials. Indeed, preliminary tests were carried out to optimize the crosslinker concentration, the employment of catalyst SHP (20% *w*/*w*, on weight of TA used, as reported in [12]) or not and the time/temperature program of the crosslinking reaction. In conclusion, the best results in terms of film stability and smoothness were the following: 10% (*w*/*w*) TA by the total weight of the polymer mixture, film drying at 40 °C for 24 h and then curing (to obtain crosslinking) at 80 °C for 2 h. No SHP was employed. Moreover, different GG/NaALG blends were evaluated and the choice of GG/NaALG ratio was carried out monitoring the oleuropein release, as reported in Figure S1. The GG:NaALG ratios equal to 1.2:0.8 and 1.6:0.4 were chosen for all the investigations. Films of GG alone and NaALG alone were also prepared but discarded, since NaALG was not considered of interest in terms of novelty, while GG produced a crumpled film after drying. When OLE-loaded films were produced, the olive extract was added as the first ingredient in the aqueous solution of the hydrogel preparation. An amount of OLE equal to 1% (*w*/*w*) over the total weight of the polymer mixture was chosen. Smooth and homogeneous film-forming solutions were cast on Petri dishes (diameter 9 cm) and dried in an oven at the above-reported optimized temperature program. The dried films were stored in desiccators with silica gel at room temperature (i.e., 20 ± 2 °C) and RH of 55 ± 2% before analyses.

### 3.3. Chemical–Physical Characterization

Hydrogel films, with and without OLE, were characterized using Fourier-transform infrared spectroscopy (FT-IR) in attenuated total reflectance mode (ATR), thermo-gravimetric analysis (TGA) and X-ray photoelectron spectroscopy (XPS).

Dry samples underwent FT-IR (ATR) analyses through a Spectrum Two PE instrument supplied by PerkinElmer, endowed with a universal ATR accessory (UATR, Single Reflection Diamond/ZnSe). For each sample, FT-IR/ATR spectra were recorded from 400 to 4000 cm^−1^ with a 4 cm^−1^ resolution.

TGA analyses were obtained through a PerkinElmer TGA-400 instrument (PerkinElmer Inc. Waltham, MA, USA). Briefly, 5–10 mg of each sample was heated in nitrogen-saturated atmosphere in the range of 30–600 °C, with a constant flow rate (20 °C/min) and a gas flow set at 20 mL/min. The TGA Pyris series software was exploited to record thermograms (TG), calculate their respective derivative curves (DTG) and for further data mining.

XPS analyses were performed on a scanning microprobe PHI 5000 VersaProbe II, purchased from Physical Electronics (Chanhassen, MN, USA). The instrument is equipped with a micro-focused monochromatized AlKα X-ray radiation source. Hydrogel films were examined in HP mode with an X-ray take-off angle of 45° (instrument base pressure ~10^−9^ mbar.). The size of the scanned area was about 1400 µm × 200 µm. Wide scans and high-resolution spectra were recorded in FAT mode for each sample, setting pass energy values equal to 117.4 eV and 29.35 eV, respectively. To fit the high-resolution spectra, the commercial MultiPak software, version 9.9.0.8, was used. Atomic percentages were inferred from peak areas, previously normalized according to MultiPak library’s sensitivity factors. Adventitious carbon C1s was set at 284.8 eV and used as reference.

### 3.4. Hydrogel Film Swelling/Deswelling, Water Holding Capacity and Gel Fraction

Dry hydrogel samples were cut into square pieces with 1 cm^2^ area, accurately weighed (m_i_^d^) and immersed in phosphate-buffered solution (PBS, pH 7.4) at 32 °C to determine the swelling kinetics up to 24 h. All the specimens were weighed after each time point (m_i_^t^). The water uptake (expressed as grams of PBS per gram of dry film) was calculated using the following formula:

Water uptake = (m_i_^t^ − m_i_^d^)/m_i_^d^ (1)

For deswelling tests, the hydrated hydrogel films were left to dry at room temperature, and the swelling percentage was monitored up to 1 h.

Moreover, for water holding capacity measurements, the hydrated hydrogel films were centrifuged in a centrifuge equipped with a basket rotor able to deliver 1400 rpm. The centrifuged samples were weighed, and the retained water was expressed as a weight percentage of the total uptake of water:

Water Holding Capacity = (m_c_ − m_d_)/(m_s_ − m_d_) × 100 (2)

where m_c_, m_s_ and m_d_ are the weights of centrifuged, swollen and dry hydrogels.

To determine the gel fraction, dry samples (1 cm^2^) were weighed accurately (m_i_) and then placed in distilled water and incubated at 50 °C for 24 h for the removal of soluble (i.e., not crosslinked) parts. The insoluble gel obtained was dried in the oven at 70 °C until constant weight (m_f_). The gel fraction was determined from the following formula:

Gel fraction (%) = m_f/_m_i_ × 100 (3)

### 3.5. Total Polyphenol Content (TPC) Determination and Radical Scavenging Activity via DPPH Assay

The TPC in OLE was determined using the Folin–Ciocâlteu colorimetric method as described by Fabiano and coworkers with some modifications [26]. Briefly, for the preparation of gallic acid (GA) stock solution for the calibration curve, 0.5 g of GA were dissolved in 10 mL ethanol and subsequently brought up to 100 mL volume with distilled water, obtaining a concentration of 5 g/L. Suitable dilutions of the GA stock solution were carried out obtaining 100, 150, 250 and 500 mg/L. An amount of 100 μL of each GA solution was added to 7.9 mL of distilled water and 500 µL of Folin–Ciocâlteu reagent. After 8 min, 1.5 mL of sodium carbonate solution (1.9 M) was added, and the absorbance was measured spectrophotometrically using a wavelength range from 690 to 810 nm and measuring the absorbance value against the blank at a wavelength of 765 nm. A triplicate of each sample was performed. The TPC in OLE and OLE-loaded hydrogel films was therefore determined via referring to the calibration curve, and the results were expressed as mg of gallic acid equivalent per gram of dry material, i.e., GAE/g. The calibration equation for GA was y = 0.0018x − 0.0511 (R^2^ = 0.9987).

OLE in vitro antioxidant activity was tested via the DPPH assay, according to the protocols described by Luo et al. [41]. Briefly, DPPH solution (100 µM) was prepared in methanol and its absorbance was measured at 517 nm. Calibration curves (r2 = 0.999) were obtained with OLE standard solutions (2 to 25 ppm). An amount of 3 mL of standard or sample solution was mixed with 1 mL of DPPH solution and their absorbance was measured at 517 nm. The radical scavenging activity percentages (% RSAs) were calculated with the following equation:

% RSA = (A_rad_ − A_S_)/A_rad_ · 100 (4)

in which A_S_ represents the sample’s absorbance, whereas A_rad_ is the absorbance of the bare DPPH. Each measurement was performed in triplicate and expressed as mean ± standard deviation. Ascorbic acid (50 µM) scavenging activity on DPPH radicals was also assessed and compared to the extract’s activity. All the assays were performed using a UV-visible Spectrophotometer UV-1900i, (Shimadzu, Milan, Italy).

### 3.6. Oleuropein Detection via HPLC

OLE was analyzed via HPLC (Prominence Series 20 with SPD-M20A PDA detector, Shimadzu, Milan, Italy) for oleuropein content, using the method previously described by Erythropel and coworkers with some modifications [42], after a preliminary filtration with 0.45 µm PTFE filters. Each sample was tested in triplicate and data were reported as mean ± standard deviation. A Shim-Pack GIST C18-AQ column (150 mm × 4.6 mm, 5 µm Shimadzu) was eluted in isocratic mode at 30 °C, 30% acetonitrile and 70% water. The effluent was monitored at 230 nm. The mobile phase flow rate was kept at 1 mL/min and samples were injected through a 20 µL injection loop. LabSolutions software was exploited to build a calibration curve (r2 0.999) with the standard compound dissolved in the mobile phase at four concentrations (1, 5, 25, 100 µg/mL).

### 3.7. In Vitro Skin Permeation Studies

A jacketed Franz diffusion cell (PermeGear Inc., SES GmbH, Bechenheim, Germany) was exploited to assess in vitro skin permeation of OLE eluting from the GG/NaALG films, following the protocol reported in a previous work [43]. Briefly, OLE-loaded hydrogels were placed in the cell donor compartment. An O-ring joint kept the film (0.6 cm^2^) on the synthetic StratM^®^ membrane (Merck KGaA, Darmstadt, Germany), characterized by skin-like porosity, diffusivity and composition. The whole assembly was fixed with a stainless-steel clamp to maintain the tight connection between the donor and receptor compartments. The Franz cell receptor chamber was filled with 5 mL PBS (pH 7.4) and continuously stirred on an ATE magnetic stirrer (VELP Scientifica Srl, Usmate, Italy). The temperature was kept constant at 32.00 ± 0.03 °C with a CD-B5 heating circulator bath (Julabo GmbH, Seelbach, Germany). At predetermined time points (15, 30, 45, 60, 120 min), PBS aliquots of 500 µL were withdrawn, replaced with fresh PBS, and analyzed via HPLC (Prominence Series 20 with SPD-M20A PDA detector, Shimadzu, Milan, Italy) for oleuropein content, as described in the HPLC Analysis Section. Additionally, at the end of the experiments, the Strat-M^®^ membrane was placed in the HPLC mobile phase overnight at 25 °C to extract and quantify the oleuropein retained by the membrane. TPC retained by the membrane was also assayed using the Folin–Ciocâlteu colorimetric method.

### 3.8. Cell Culture

Normal human dermal fibroblasts (NhDFs) were cultured in high-glucose Dul-becco’s Modified Eagle Medium (HG-DMEM; Corning Inc., Corning, NY, USA), supplemented with 10% fetal bovine serum (Corning Inc.), 1% L-glutamine (Thermo Fisher Scientific, Waltham, MA, USA) and 1% penicillin/streptomycin (Thermo Fisher Scientific) at 37 °C with 5% CO_2_. For all experiments, NhDFs were seeded at cell density 1.8 × 10^4^ cells/cm^2^.

To treat cells, the different hydrogels (GG1.2/NaALG0.8, GG1.6/NaALG0.4, GG1.2/NaALG0.8-OLE, GG1.6/NaALG0.4-OLE) were incubated in normal medium for 24 h at 37 °C. To simulate photoaging effects, cells were irradiated from above using a Philips Original Home Solarium sun lamp (model HB 406/A; Philips, Groningen, Holland) equipped with a 400 W ozone-free Philips HPA lamp, UV type 3, delivering a flux of 45 mW/cm^2^ between 300 and 400 nm, at a distance of 20 cm from the samples. The dose of UVA (~13.5 J/cm^2^) received from above by the samples was measured with a UV Power Pack Radiometer (EIT Inc., Sterling, VA, USA), while the emission spectrum was checked using a StellarNet portable spectroradiometer (Tampa, FL, USA).

For irradiation, the cells grown on 96-well culture plates were washed and covered with a thin layer of PBS. To limit evaporation during UVA exposure, a quartz plate of the same dimension as the culture dish was used as cover. The dish was then placed on a brass block embedded on ice and exposed to the UVA source as described above. For the negative control, the cells were not exposed to UVA.

After UVA exposure, normal medium was replaced with the conditioned media and NhDFs were incubated for further 24 h. Normal medium and medium containing 55 μg/mL OLE were used as controls.

### 3.9. Cell Viability

UVA-irradiated and non-irradiated fibroblasts were tested for viability after 24 h treatments with the different media. Cells were incubated with AlamarBlue^TM^ (Invitrogen, Carlsbad, CA, USA) for 4 h, before reading at 570 nm, with 600 nm as reference wavelength, using a MultiskanGO plate reader (Thermo Fisher Scientific).

### 3.10. F-Actin Staining and Mean Area Measuring

NhDFs were seeded on round 13 mm microscope slides and treated as described above. To underline cytoskeletal F-actin, cells were fixed with 4% paraformaldehyde in PBS pH 7.4 for 30 min at 4 °C, washed with PBS and permeabilized with 0.1% Triton X-100 in PBS at RT for 30 min. After washing, NhDFs were incubated with TRITC-labelled phalloidin (dilution 1:500; Sigma-Aldrich, St. Louis, MO, USA) for 1 h at RT, and then with Hoechst 33342 (dilution 1:10,000; Thermo Fisher Scientific). Slides were mounted with Vectashield mounting medium (Vector Labs, Burlingame, CA, USA) and evaluated under fluorescent microscope Eclipse 600 (Nikon, Milan, Italy). NIS-Elements microscope imaging software (Nikon) was used to capture images. Eight 20× images were taken for each experimental condition and processed within FIJI (https://imagej.net/software/fiji/downloads (accessed on 28 May 2020)) to calculate the percentage of mean cell area, normalizing on the number of nuclei.

### 3.11. Morphology and Intracellular Structure in 4D Live Cell Imaging

The morphology and subcellular structure of NhDFs were observed in real time through 4D live-cell imaging with holotomographic microscopy (HTM) using the 3D Cell Explorer-Fluo (Nanolive, Ecublens, Switzerland). NhDFs were seeded into µ-Dishes 35 mm (Ibidi, Gräfelfing, Germany) and UVA irradiated as explained above. Cells were then treated with normal medium or conditioned media (GG1.2/NaALG0.8-OLE and GG1.6/NaALG0.4-OLE) and immediately incubated for 24 h with a top-stage incubator (Okolab, Pozzuoli, Italy) at 37 °C, humidity and 5% CO_2_ throughout image acquisitions. Images were captured every 3 min for 24 h. Live Cell Imaging Software STEVE (Nanolive, Ecublens, Switzerland), which controls the HTM microscope, was used to obtain RI volumes and convert them into TIFF format.

The 3D RI volumes in TIFF format were then processed within FIJI and saved as a video in AVI format.

### 3.12. Western Blotting

NhDFs were seeded into 6-well plates and treated as stated before. After UVA and media treatments, cells were washed with PBS and detached using Trypsin 1× to collect pellets. For protein extraction, pellets were lysed in Denaturing Lysis Buffer (50 mM Tris-HCl, 150 mM NaCl, 1% Triton X-100, 0.1% Sodium Dodecyl Sulfate), supplemented with 1 mM PMSF, protease inhibitors (Sigma-Aldrich) and PhosStop (Roche, Basel, Switzerland). The supernatants were collected after centrifugation at 12,000× g for 10 min at 4 °C.

DC protein assay (Bio-Rad, Hercules, CA, USA) was performed to measure the total protein amount, and protein samples were prepared to load 10 µg of protein for each sample. Samples were prepared using NuPAGE™ LDS Sample Buffer 4× (Invitrogen) and fractionated in Bolt™ 4 to 12% Bis-Tris gels (Invitrogen). Proteins were electrophoretically transferred to 0.2 μm nitrocellulose membranes (Bio-Rad, Hercules, CA, USA). Membranes were incubated with 5% milk in Tris-Buffered Saline with 0.1% Tween 20 (TBS-T) to block non-specific sites and then with primary antibodies in 5% milk in TBS-T at 4 °C overnight (anti-COL1A2: dilution 1:1000, cat. n. 14695-1-AP, Proteintech, Manchester, UK; anti-Decorin: dilution 1:1000, cat. n. Ab175404, Abcam, Cambridge, UK; Stress Defense cocktail (Catalase, SOD1): dilution 1:1000, cat. n. Ab179843, Abcam). After washes with TBS-T, the membranes were incubated with secondary antibodies anti-mouse (dilution 1:30,000, Bethyl laboratories, Waltham, MA, USA) and anti-rabbit (dilution 1:10,000, Invitrogen) conjugated with horseradish peroxidase. Detection of antibody binding was performed with Clarity Western ECL Substrate (Bio-Rad), and images were acquired with Alliance Mini HD9 (Uvitec, Cambridge, UK). Densitometric analysis was performed with FIJI software and protein expression was normalized on Ponceau staining.

### 3.13. RT-PCR

NhDFs were seeded into 6-well plates. After UVA and media treatments, cells were washed with PBS and detached using Trypsin 1× to collect pellets. For RNA extraction, pellets were incubated o.n. with TRIzol^TM^ reagent (Invitrogen) and processed according to manufacturer’s instructions. For each sample, 300 ng RNA were retrotranscripted with SuperScript™ IV VILO™ MasterMix with ezDNase (Invitrogen).

qRT-PCR was performed to amplify the obtained cDNA using PowerUp™ SYBR™ Green Master Mix (Applied Biosystems, Waltham, MA, USA). IL-6, TNF-α and GAPDH primers were used (Table 4) [44]. The mRNA expression was calculated using the 2^−ΔΔCt^ method and shown as Relative Expression.

### 3.14. Statistical Analysis

The statistical analysis was performed using GraphPad Prism 9 (GraphPad Software, San Diego, CA, USA). Results were analyzed using one-way ANOVA tests, and multiple comparisons among the groups were evaluated using Tukey’s test. Statistical significance was considered at *p* < 0.05.

## 4. Conclusions

In this work, GG/NaALG hydrogel films loaded with an oleuropein-rich olive leaf extract were proposed for potential use as facial masks, with the aim to counteract the harmful effects of UVA radiation on the skin. FTIR/ATR, XPS, swelling/deswelling, gel fraction, water holding capacity and thermal properties were thoroughly studied and discussed. The total polyphenol content and antioxidant activity were investigated, and the ability of the hydrogel films to convey oleuropein to the skin was also evaluated using a Franz cell apparatus. Finally, we demonstrated that the addition of OLE into the developed hydrogels was useful to counteract damage caused by UVA exposure. Since UVA-induced damage is known to be mainly mediated via ROS production, we propose that the enriched hydrogel GG1.2NaALG0.8-OLE could be the most effective one to contrast oxidative stress and, consequently, to reduce inflammation. In addition, this hydrogel stimulated the expression of ECM proteins fundamental for restoring normal dermis composition. Moreover, GG1.6NaALG0.4-OLE helped restore normal fibroblast morphology, which is normally altered in the photoaged dermis.

## Figures and Tables

**Table 1:** Surface atomic compositions of OLE, GG, NaALG and films with and without OLE.

Element	OLE	GG	NaALG	GG1.2NaALG0.8	GG1.6NaALG0.4	GG1.2NaALG0.8-OLE	GG1.6NaALG0.4-OLE
C1s	63.8	56.9	56.1	62.0	64.1	60.0	61.1
O1s	33.6	40.1	38.0	35.1	33.2	37.3	36.0
Si2p	3.0	-	-	-	-	0.3	0.3
N1s	-	1.9	-	1.1	1.2	0.9	1.0
S2p	-	0.5	-	-	-	-	-
Na1s	-	0.3	5.9	1.5	1.1	1.5	1.1
Ca2p	-	0.3	-	-	-	-	-

**Table 2:** Attributions, BEs and atomic percentages of the peaks reported in the curve fittings of Figure 3 (BEs uncertainty: ±0.2 eV).

Attribution	GG1.2NaALG0.8	GG1.6NaALG0.4	GG1.2NaALG0.8-OLE	GG1.6NaALG0.4-OLE
	BE(eV)/At%			
CHx	284.8/37.8	284.8/31.1	284.8/38.7	284.8/27.3
C–COOR	-	-	285.5/2.6	285.5/2.4
C–OR	286.4/42.5	286.4/49.2	286.4/40.9	286.4/44.8
O–C–O or C=O	287.7/12.3	287.7/14.2	287.8/10.8	287.8/17.3
COOH(R)	288.7/7.5	288.7/5.4	288.8/7.3	289.0/8.1

**Table 3:** Gel fraction and WHC of GG1.2NaALG0.8, GG1.6NaALG0.4, GG1.2NaALG0.8-OLE and GG1.6NaALG0.4-OLE hydrogel films. Data expressed as mean ± standard deviation over three replicates.

Sample	Gel Fraction (%)	WHC (%)
GG1.2NaALG0.8	60.2 ± 1.1	70 ± 3
GG1.6NaALG0.4	67.7 ± 1.3	82 ± 4
GG1.2NaALG0.8-OLE	59 ± 2	76 ± 2
GG1.6NaALG0.4-OLE	66.1 ± 1.8	80.5 ± 1.5

**Table 4:** Sequences of primers used for RT-PCR analysis.

Gene	Sequence
IL-6 Fw	5′-CATTTGTGGTTGGGTCAGGG
IL-6 Rv	5′-CCAGAGCTGTGCAGATGAGT
TNF-α Fw	5′-GGTGCTTGTTCCTCAGCCTC
TNF-α Rv	5′-AGATGATCTGACTGCCTGGG
GAPDH Fw	5′-GTCTCCTCTGACTTCAACAGCG
GAPDH Rv	5′-ACCACCCTGTTGCTGTAGCCAA

## Data Availability

Data are available from the authors on request.

## Supplementary Materials

The following supporting information can be downloaded at: https://www.mdpi.com/article/10.3390/molecules28114352/s1, Figure S1: Percentage of oleuropein released in PBS after 24 h in function of GG amount present in the GG/NaALG films. Figure S2: TGA (a) and DTGA (b) traces of GG and NaALG polymers. Figure S3: C1s high-resolution spectra and relevant curve fittings recorded on OLE, GG and NaALG. Attributions, BE values and atomic percentages are reported in the table (BEs uncertainty: ± 0.2 eV). Figure S4: Morphological evaluation of NhDFs. F-actin staining of not-irradiated and not-treated (Not-irradiated), not-treated (CTR) and OLE-treated (OLE) NhDFs at 24 h after irradiation (magnification: 20×; scale bar: 50 μm). Video S1: 4D live imaging of NhDFs for 24 h. Video S2: 4D live imaging of NhDFs for 24 h after UVA irradiation. Video S3: 4D live imaging of NhDFs for 24 h after UVA irradiation and GG1.2NaALG0.8-OLE treatment. Video S4: 4D live imaging of NhDFs for 24 h after UVA irradiation and GG1.6NaALG0.4-OLE treatment.

## Sample Availability

Samples of the compounds are available from the authors.
