# Supplementary material for: Oleuropein-Rich Gellan Gum/Alginate Films as Innovative Treatments against Photo-Induced Skin Aging
Source: Molecules. 2023 May 25;28(11):4352. doi: 10.3390/molecules28114352 (PMC10254495; doi:10.3390/molecules28114352)
Supplement: Supplementary file 1 [file molecules-28-04352-s001.zip › Supplementary Material.pdf]

Supplementary Material

Oleuropein-rich Gellan Gum/Alginate films as innovative treatments against photo-induced skin aging

Francesco Busto[1, 2#], Caterina Licini[3#], Alessia Luccarini[4], Elisabetta Damiani[4], Monica Mattioli Belmonte[2,3], Stefania Cometa[5*,] Elvira De Giglio[1,2*]

1 Department of Chemistry, University of Bari, Via Orabona 4, 70126 Bari, Italy; f.busto3@studenti.uniba.it (FB)
2 INSTM, National Consortium of Materials Science and Technology, Via G. Giusti 9, 50121 Florence, Italy
3 DISCLIMO, Università Politecnica delle Marche, Via Tronto 10/a 60126 Ancona, Italy; c.licini@staff.univpm.it (C.L.); m.mattioli@staff.univpm.it (M.M.-B.)
4 DISVA, Università Politecnica delle Marche, Via Brecce Bianche 60131 Ancona, Italy; a.luccarini@pm.univpm.it (A.L.); e.damiani@univpm.it (E.D.)
5 Jaber Innovation s.r.l., Via Calcutta 8, 00144 Rome, Italy
* Correspondence: stefania.cometa@jaber.it (S.C.) elvira.degiglio@uniba.it (E.D.G.)
# These authors contributed equally to this work.

GG/NaALG hydrogel film preparation

To prepare GG/NaALG hydrogel films, different ratios of GG:NaALG were tested, maintaining the sum of the two polymer percentages equal to 2% (w/v) i.e., 0.4:1.6, 0.8:1.2, 1:1, 1.2: 0.8 and 1.6: 0.4. The choice of the ideal GG:NaALG ratios was carried out monitoring the oleuropein release, as shown in Figure 1s.

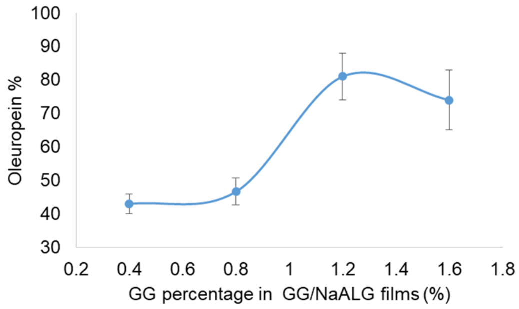

Figure s1: Percentage of oleuropein released in PBS after 24 hours in function of GG amount present in the GG/NaALG films.

Moreover, the film crosslinking procedure was optimized evaluating different variables such as temperature, times, employment of the catalyst and TA concentrations. In particular, the following curing temperature/times conditions were tested: 80 for 2 hours or 140°C for 30 minutes. Moreover, the employment or not of a catalyst SHP (20% w/w, on weight of TA used) was also evaluated, as well as the choice of TA concentration. From the analysis of the FTIR/ATR spectra and from swelling and stability tests of the obtained films, it was concluded that the best crosslinking conditions resulted those reported in Section 3.2 of the manuscript.

TGA analysis

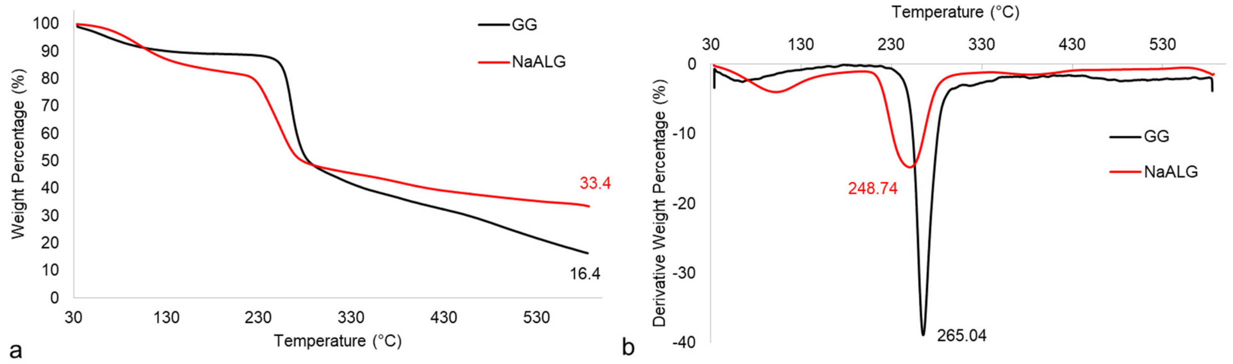

Figure s2: TGA (a) and DTGA (b) traces of GG and NaALG polymers.

XPS analysis

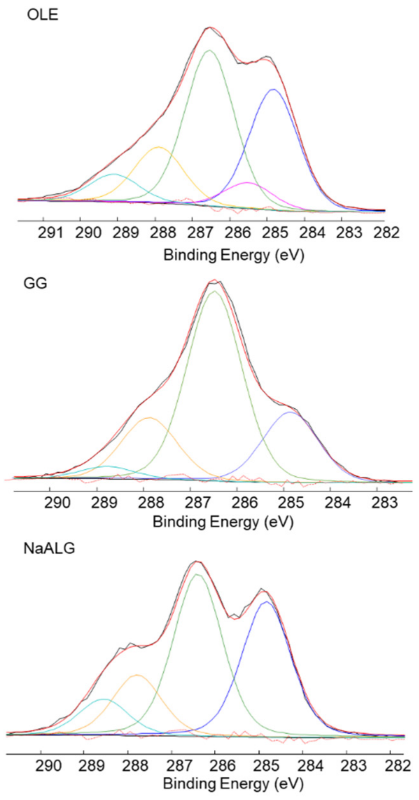

Attribution	***OLE***	***GG***	***NaALG***
	BE(eV)/ At %		
CHx	284.8/31.3	284.8/21.4	284.8/34.5
C-COOR	285.5/6.9	-	--
C-OR/286.5	286.5/40.4	286.5/56.6	286,4/41.0
O-C-O or C=O	287.9/14.5	287.9/18.4	287.8/15.4
COOR/288.6-289.0	289,0/7.1	288.8/3.6	288.6/9.1

Figure s3: C1s high-resolution spectra and relevant curve fittings recorded on OLE, GG and NaALG. Attributions, BE values and atomic percentages are reported in the table (BEs uncertainty: ± 0.2 eV).

Morphological features and live cell imaging in NhDFs

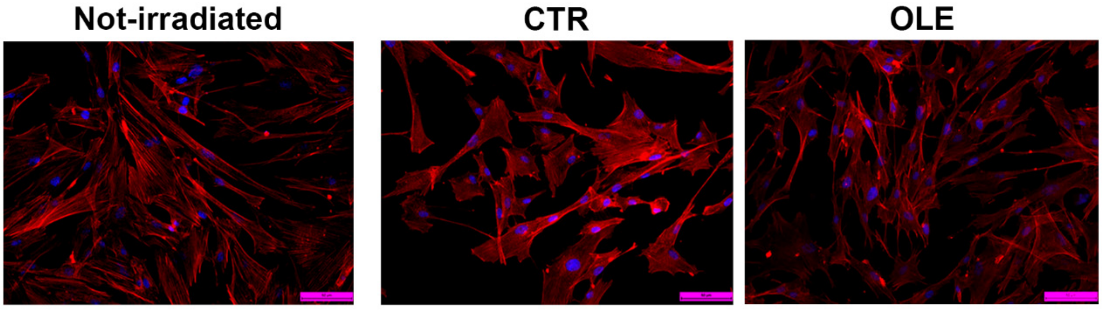

Figure s4: Morphological evaluation of NhDFs. F-actin staining of not-irradiated and not-treated (Not-irradiated), not-treated (CTR) and OLE-treated (OLE) NhDFs at 24 h after irradiation (magnification: 20x; scale bar: 50 µm).

Supplementary video captions:

Suppl. Video S1: 4D live imaging of NhDFs for 24 h.

Suppl. Video S2: 4D live imaging of NhDFs for 24 h after UVA irradiation.

Suppl. Video S3: 4D live imaging of NhDFs for 24 h after UVA irradiation and GG1.2NaALG0.8-OLE treatment.

Suppl. Video S4: 4D live imaging of NhDFs for 24 h after UVA irradiation and GG1.6NaALG0.4-OLE treatment.
